# High versus Standard Intensity of Thromboprophylaxis in Hospitalized Patients with COVID-19: A Systematic Review and Meta-Analysis

**DOI:** 10.3390/jcm10235549

**Published:** 2021-11-26

**Authors:** Anastasios Kollias, Konstantinos G. Kyriakoulis, Ioannis P. Trontzas, Vassiliki Rapti, Ioannis G. Kyriakoulis, Christina A. Theochari, Evangelos Dimakakos, Garyphallia Poulakou, Konstantinos Syrigos

**Affiliations:** Third Department of Medicine, National and Kapodistrian University of Athens, School of Medicine, Sotiria Hospital, Athens 11527, Greece; konkyriakoulis@gmail.com (K.G.K.); john-tron@hotmail.com (I.P.T.); vassiarapti@gmail.com (V.R.); ioannis.kyriakoulis@gmail.com (I.G.K.); xristinath13@gmail.com (C.A.T.); edimakakos@yahoo.gr (E.D.); gpoulakou@gmail.com (G.P.); ksyrigos@med.uoa.gr (K.S.)

**Keywords:** anticoagulation, COVID-19, COVID-19 therapeutics, dose, meta-analysis, mortality, thromboprophylaxis, treatment

## Abstract

Thromboprophylaxis in hospitalized patients with COVID-19 has been associated with a survival benefit and is strongly recommended. However, the optimal dose of thromboprophylaxis remains unclear. A systematic review and meta-analysis (PubMed/EMBASE) of studies comparing high (intermediate or therapeutic dose) versus standard (prophylactic dose) intensity of thrombo-prophylaxis with regard to outcome of hospitalized patients with COVID-19 was performed. Randomized and non-randomized studies that provided adjusted effect size estimates were included. Meta-analysis of 7 studies comparing intermediate versus prophylactic dose of thromboprophylaxis (2 randomized and 5 observational, *n* = 2009, weighted age 61 years, males 61%, ICU 53%) revealed a pooled adjusted relative risk (RR) for death at 0.56 (95% confidence intervals (CI) 0.34, 0.92) in favor of the intermediate dose. For the same comparison arms, the pooled RR for venous thromboembolism was 0.84 (95% CI 0.54, 1.31), and for major bleeding events was 1.63 (95% CI 0.79, 3.37). Meta-analysis of 17 studies comparing therapeutic versus prophylactic dose of thromboprophylaxis (2 randomized and 15 observational, *n* = 7776, weighted age 64 years, males 54%, ICU 21%) revealed a pooled adjusted RR for death at 0.73 (95% CI 0.47, 1.14) for the therapeutic dose. An opposite trend was observed in the unadjusted analysis of 15 observational studies (RR 1.24 (95% CI 0.88, 1.74)). For the same comparison arms, the pooled RR for venous thromboembolism was 1.13 (95% CI 0.52, 2.48), and for major bleeding events 3.32 (95% CI 2.51, 4.40). In conclusion, intermediate compared with standard prophylactic dose of thromboprophylaxis appears to be rather safe and is associated with additional survival benefit, although most data are derived from observational retrospective analyses. Randomized studies are needed to define the optimal thromboprophylaxis in hospitalized patients with COVID-19.

## 1. Introduction

Venous thromboembolic events (VTE) constitute one of the major complications of critical COVID-19 and are associated with adverse outcome [1,2,3]. Furthermore, thrombosis and microvascular disease in small pulmonary blood vessels and capillaries has been found in several autopsy studies of patients whose cause of death was COVID-19 [4]. Moreover, the administration of thromboprophylaxis in hospitalized patients with COVID-19 has been associated with survival benefit [5,6]. Based on such available evidence, current guidelines recommend thromboprophylaxis in all hospitalized patients with COVID-19, mainly in the form of prophylactic dose of low molecular weight heparin (LMWH) [7].

However, some of these guidelines qualify a higher (intermediate) than prophylactic dose of anticoagulation in patients with severe COVID-19 and increased thromboembolic risk [7], despite the fact that the latter recommendation represents mainly expert opinion rather than evidence [6]. Indeed, the available evidence is weak since this is derived mainly from observational studies, where the selection of higher versus prophylactic doses of anticoagulation has been decided for patients with critical disease. However, in these patients, the benefit of this strategy might be blunted by the adverse prognosis of severe COVID-19. It might be argued that the benefit of the anticoagulation strategy is gained only with early administration and before the establishment of irreversible lung damage [8]. Recent randomized controlled trials provide higher quality data but their findings are controversial, mainly due to the heterogeneity in the characteristics of the study population (general ward or intensive care unit (ICU) patients, degree of COVID-19 severity), as well as the time of the initiation of thromboprophylaxis, which might affect the outcome [9,10,11,12,13].

The aim of this systematic review and meta-analysis was to assess the risk of in-hospital mortality in hospitalized patients with COVID-19 receiving high (intermediate or therapeutic) versus prophylactic doses of thromboprophylaxis, by using data from randomized or observational studies providing adjusted analyses.

## 2. Materials and Methods

### 2.1. Registration and Reporting

This systematic review and meta-analysis was performed according to preferred reporting items for systematic reviews and meta-analyses (PRISMA) guidelines [14]. The PRISMA 2020 checklist for the present meta-analysis is presented in Supplementary File, Table S1. The PRISMA 2020 abstracts checklist is presented in Supplementary File, Table S2. The protocol was registered in the PROSPERO international prospective register of systematic reviews (CRD42021286921).

### 2.2. Search Strategy

A systematic search of PubMed and EMBASE databases was performed until October 1st, 2021 using the following search algorithm: (“coronavirus 2019” OR “2019-nCoV” OR “SARS-CoV-2” OR “COVID-19” OR COVID OR COVID19) AND (anticoag* OR dosing OR dose OR intensity OR thromboprophyla* OR intermediate OR prophylactic) AND (thrombotic OR thrombosis OR “deep vein” OR “pulmonary embolism” OR thromboemboli* OR death OR mortality OR fatal OR survival OR outcome OR intubation OR bleed* OR hemorrhag* OR haemorrhag*). Articles were also identified from reference lists of previously conducted relevant systematic reviews and meta-analyses and relevant papers and websites through snowball procedure. 

### 2.3. Study Selection

The study selection was performed independently by five investigators (K.G.K., I.P.T., V.R., I.G.K., and C.A.T.). Discrepancies were resolved by consensus with a senior author (A.K.). Eligible studies were full-text articles in English language including ≥20 patients (not case series) that had either a randomized design or were observational but reported both unadjusted (or provided the number of events in each group) and adjusted hazard or odds ratios or relative risks (RR) for mortality (primary endpoint) for high (either intermediate or therapeutic dose) versus standard (prophylactic dose) intensity of thromboprophylaxis in hospitalized COVID-19 patients. No restriction was applied concerning the type of anticoagulant used. Doses were defined and categorized according to each study definitions as prophylactic, intermediate, and therapeutic. 

### 2.4. Data Extraction

Five investigators (K.G.K., I.P.T., V.R., I.G.K., and C.A.T.) extracted and tabulated, independently, data concerning study design, main characteristics of included populations, and that regarding the primary (adjusted hazard/odds ratio or RR for mortality) and secondary (VTE and bleeding events) outcomes of interest. 

### 2.5. Risk of Bias Assessment

The risk of bias was assessed in terms of selection of patients, exposure measurement, confounding factors identification, outcome measurement, methodology, and analysis, independently, by five investigators (K.G.K., I.P.T., V.R., I.G.K., and C.A.T.). Checklists for cohort studies and for randomized controlled trials from Joanna Briggs Institute Critical Appraisal Tools were used [15]. Observational studies fulfilling ≥8 and randomized controlled trials fulfilling ≥9 of the quality domains were deemed as low risk of bias.

### 2.6. Certainty (Confidence) of the Outcome 

The certainty of the body of evidence for the outcome of death was independently assessed by two investigators (K.G.K. and A.K.) using the GRADE approach (grading of recommendations assessment, development and evaluation) described in Chapter 14 of *The Cochrane Handbook for Systematic Reviews of Interventions* [16]. The certainty of evidence was deemed as high, moderate, low, or very low, depending on factors that either decrease the confidence of the outcome—such as the risk of bias, the publication bias, the inconsistency, the indirectness, and the imprecision of results—or factors that increase the certainty—such as the large effect size, the dose response, and the effect of plausible residual confounding [17].

### 2.7. Statistical Analysis

Meta-analysis was performed using the Stata/SE 11 (Texas) software. Logarithms of adjusted RR and corresponding standard errors were used for the analysis (fixed-effects meta-analysis when I^2^ statistic value <50%). Odds ratios were converted to RR according to appropriate formula [18]. Hazard ratios were treated as RR. Results were graphically displayed as forest plots. Sensitivity analysis was performed to investigate the impact of different thromboprophylaxis doses in studies conducted exclusively in ICU or not (general wards or mixed settings). Meta-regression analysis was performed for assessing associations of the RR for mortality with mean age, mean d-dimer value, and percentage of males, diabetics, and ICU patients. Mean values of subgroups were combined where feasible [19]. Median (interquartile range) values were converted to mean values (standard deviation) using appropriate formulas [20]. Heterogeneity was tested using I^2^ statistics. Publication bias was assessed by inspecting funnel plots, as well as Egger’s test (linear regression method) and Begg’s test (rank correlation method) [21,22]. Two-sided *p* values of <0.05 were considered statistically significant. Missing information was retrieved after communication with the corresponding authors.

## 3. Results

### 3.1. Literature Search and Inclusion of Studies

Among the 8318 articles initially retrieved through our literature search, 21 fulfilled the inclusion criteria and were included in our analysis [11,12,23,24,25,26,27,28,29,30,31,32,33,34,35,36,37,38,39,40,41]. The PRISMA 2020 flow diagram for systematic reviews and meta-analyses study selection is presented in Supplementary File, Figure S1. A total of 7 studies reported data for intermediate versus prophylactic dose [11,27,28,29,30,34,35], while 17 studies reported data for therapeutic versus prophylactic dose of thromboprophylaxis [12,23,24,25,26,29,31,32,33,34,35,36,37,38,39,40,41]. Three studies contributed data for both intermediate versus prophylactic and therapeutic versus prophylactic dose analyses [29,34,35]. The main characteristics of the included studies are shown in Table 1. A list of the adjustment variables included in the multivariate analyses of the observational studies is presented in Supplementary File, Table S3. 

### 3.2. Data Synthesis

#### 3.2.1. Intermediate versus Prophylactic Dose of Anticoagulation

**Figure 1:**
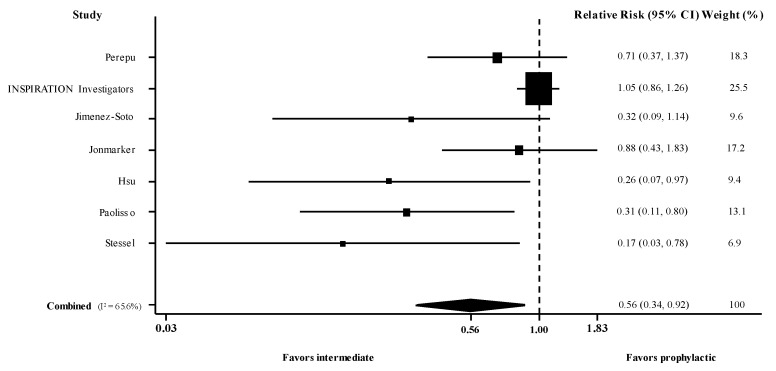
Forest plot of adjusted risk ratios for death in hospitalized patients with COVID-19 administered intermediate versus prophylactic dose of thromboprophylaxis. CI, confidence intervals; I^2^, test for heterogeneity.

There were 2 randomized [11,30] and 5 observational studies [27,28,29,34,35] (*n* = 2009, weighted age 61 years, males 61%, ICU 53%) that reported the RR for death in patients with COVID-19 administered intermediate versus prophylactic dose of thromboprophylaxis. Meta-analysis of these 7 studies (use of adjusted estimates for the non-randomized) revealed a pooled adjusted RR for death of 0.56 (95% confidence intervals [CI] 0.34, 0.92; I^2^ 66%) (Figure 1). Meta-analysis of the 5 observational studies [27,28,29,34,35] showed pooled unadjusted RR at 0.45 (95% CI 0.29, 0.69; I^2^ 28%), whereas the adjusted pooled RR remained the same at 0.45 (95% CI 0.28, 0.72; I^2^ 36%). 

Regarding the secondary outcomes, meta-analysis of 6 studies [11,27,29,30,34,35] revealed a pooled unadjusted RR for VTE at 0.84 (95% CI 0.54, 1.31; I^2^ 0%) and meta-analysis of 7 studies [11,27,28,29,30,34,35] revealed a pooled RR for major bleeding events at 1.63 (95% CI 0.79, 3.37; I^2^ 0%) for intermediate versus prophylactic dose of thromboprophylaxis. 

#### 3.2.2. Therapeutic versus Prophylactic Dose of Thromboprophylaxis

**Figure 2:**
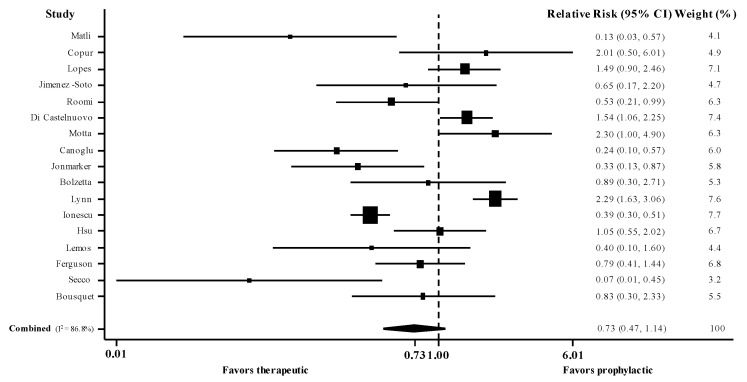
Forest plot of adjusted risk ratios for death in hospitalized patients with COVID-19 administered therapeutic versus prophylactic dose of thromboprophylaxis. CI, confidence intervals; I^2^, test for heterogeneity.

There were 2 randomized [12,33] and 15 observational studies [23,24,25,26,29,31,32,34,35,36,37,38,39,40,41] (*n* = 7776, weighted age 64 years, males 54%, ICU 21%) that reported the RR for death in patients with COVID-19 administered therapeutic versus prophylactic dose of thromboprophylaxis. Meta-analysis of these 17 studies (use of adjusted estimates for non-randomized) revealed a pooled adjusted RR for death at 0.73 (95% CI 0.47, 1.14; I^2^ 87%) (Figure 2). Meta-analysis of the 15 observational studies showed pooled unadjusted RR for death at 1.24 (95% CI 0.88, 1.74; I^2^ 87%), whereas the adjusted pooled RR was 0.71 (95% CI 0.44, 1.15; I^2^ 88%).

Regarding the secondary outcomes, meta-analysis of 6 studies [12,29,31,33,34,35] revealed a pooled unadjusted RR for VTE at 1.13 (95% CI 0.52, 2.48; I^2^ 58%) and meta-analysis of 9 studies [24,25,29,31,33,34,35,37,38] revealed a pooled unadjusted RR for major bleeding events at 3.32 (95% CI 2.51, 4.40; I^2^ 0%) for therapeutic versus prophylactic dose of thromboprophylaxis.

### 3.3. Sensitivity and Meta-Regression Analyses

In sensitivity analyses, meta-analysis of 3 studies conducted exclusively in ICU [11,27,35] revealed a pooled adjusted RR for death in patients with COVID-19 administered intermediate versus prophylactic dose of thromboprophylaxis at 0.80 (95% CI 0.43, 1.50; I^2^ 59%), whereas meta-analysis of 4 studies conducted in general wards or mixed settings (general ward/ICU) [28,29,30,34] revealed a pooled adjusted RR at 0.47 (95% CI 0.29, 0.75; I^2^ 12%). Meta-analysis of 3 studies conducted exclusively in ICU [12,35,37] revealed a pooled adjusted RR for death in patients with COVID-19 administered therapeutic versus prophylactic dose of thromboprophylaxis at 0.58 (95% CI 0.35, 0.94; I^2^ 24%), whereas meta-analysis of 14 studies conducted in general wards or mixed settings (general ward/ICU) [23,24,25,26,29,31,32,33,34,36,38,39,40,41] revealed a pooled adjusted RR at 0.79 (95% CI 0.48, 1.30; I^2^ 89%).

Multivariate meta-regression analysis did not reveal any significant associations between RR for death for intermediate versus prophylactic dose and mean age (regression coefficient (RC) −0.04, 95% CI −0.32, 0.23), percentage of male (RC 0.02, 95% CI −0.15, 0.19) and diabetic (RC 0.02, 95% CI −0.16, 0.19) patients. In addition, there was no association between the RR and the mean d-dimer value (RC 0.001, 95% CI −0.002, 0.004), but there was a trend for lower RR with lower percentage of ICU patients (RC 0.01, 95% CI −0.0004, 0.02; *p* = 0.06) (these variables were examined in univariate meta-regression analyses due to insufficient observations). Multivariate meta-regression analysis did not reveal any significant associations between RR for death for therapeutic versus prophylactic dose and mean age (RC 0.03, 95% CI −0.31, 0.37), percentage of male (RC −0.009, 95% CI −0.27, 0.25), diabetic (RC 0.14, 95% CI −0.68, 0.95), and ICU (RC 0.004, 95% CI −0.06, 0.07) patients, as well as with the mean d-dimer value (RC −0.001, 95% CI −0.007, 0.005).

### 3.4. Risk of Bias, Publication Bias, and Certainty of the Evidence Assessment

The assessment of the risk of bias of the included studies comparing intermediate or therapeutic versus prophylactic dose of thromboprophylaxis is presented in Supplementary File, Tables S4–S6. All studies were deemed as low risk of bias, mainly due to their randomized design or the strict inclusion criteria of the observational studies providing adjusted analyses for several confounders. 

Egger’s test and Begg’s funnel plots revealed a small study effect (*p* = 0.02 and 0.05, respectively) for intermediate versus prophylactic dose but not for therapeutic versus prophylactic (*p* = 0.61 and 0.07, respectively) (Supplementary File, Figure S2).

The certainty of the evidence on the outcome of death was low in terms of a beneficial effect of intermediate or therapeutic versus prophylactic dose of thromboprophylaxis in hospitalized COVID-19 patients (Supplementary File, Table S7).

## 4. Discussion

This meta-analysis summarized the available evidence on the efficacy and safety of enhanced (intermediate or therapeutic) versus standard (prophylactic) dose of thromboprophylaxis in hospitalized patients with COVID-19. The main findings include the following: (i) intermediate dose of thromboprophylaxis seems to be associated with additional benefit in terms of survival compared with prophylactic dose; (ii) therapeutic versus prophylactic dose of thromboprophylaxis seems to be associated with an increased risk for major hemorrhage, whereas the benefit in terms of survival is questionable; (iii) the evidence is mainly derived from observational studies; (iv) LMWH is the main anticoagulant that has been used for thromboprophylaxis. 

The majority of the available guidance documents recommend standard prophylactic low dose of thromboprophylaxis in all hospitalized patients; however, higher doses can be selectively recommended on an individualized basis for patients at high or very high thrombotic risk, provided they also have a low risk of bleeding [7]. The available evidence, mainly derived from observational studies, is heterogeneous regarding the beneficial role of higher doses since the latter are administered in patients with critical disease and unfavorable prognostic factors [7,42]. Recent randomized studies have been published providing a high level of evidence, but their findings seem to be heterogeneous as well [9,10,11,12,13,30,33]. The current meta-analysis included only studies that were either randomized or observational that provided adjusted effect size estimates for high versus standard dose of thromboprophylaxis, which might mitigate the above-mentioned methodological challenges. 

The present analysis included mainly observational studies. Most studies used LMWH for thromboprophylaxis. Intermediate compared with prophylactic dose appeared to be associated with an about 45% decrease in mortality. A trend towards increased incidence of major bleeding events with intermediate dose was observed; however, this did not reach statistical significance. On the other hand, therapeutic dose was not observed to show a significant effect on reducing mortality compared to prophylactic dose. However, opposite trends were observed in the unadjusted and adjusted analyses. More specifically, a trend towards harm was observed in the unadjusted analysis (RR 1.24), whereas a trend towards benefit was observed in the adjusted analysis, including data from the same studies (RR 0.71). This finding highlights the indication bias of the included observational studies: higher doses were selectively administered in patients with higher risk for severe disease due to their baseline risk factors and/or high levels of indices of COVID-19 severity. Thus, their adverse prognosis might mitigate the benefit of this strategy or even mislead to a link between high dose and mortality. Adjustment for appropriate confounders seems to be necessary with this respect; however, randomized trials are the most appropriate studies for providing the highest level of evidence.

Evidence from randomized trials has become available lately; however, their findings should be interpreted with caution. In the Intermediate versus Standard-Dose Prophylactic Anticoagulation in Critically-ill Patients with COVID-19: An Open Label Randomized Controlled Trial (INSPIRATION), 562 ICU patients were randomized to receive either intermediate or therapeutic dose of thromboprophylaxis [11]. Intermediate dose did not offer significant benefits either in the primary composite outcome (acute VTE, arterial thrombosis, treatment with extracorporeal membrane oxygenation, or all-cause mortality), or in each one of its components. It should be noticed however, that all patients had critical disease and randomization was performed after a median of 13 days from symptoms onset, with no available data regarding their previous anticoagulation regimen [8,11]. A plausible explanation could be that microvascular disease in small pulmonary blood vessels and capillaries may have already been established in critically ill patients, rendering intensified anticoagulation non-efficacious at this timepoint [43]. Similarly, a landmark study including data of 1098 critically ill patients from 3 different platforms (Randomized, Embedded, Multifactorial Adaptive Platform Trial for Community-Acquired Pneumonia (REMAP-CAP); A Multicenter, Adaptive, Randomized Controlled Platform Trial of the Safety and Efficacy of Antithrombotic Strategies in Hospitalized Adults with COVID-19 (ACTIV-4a); The Antithrombotic Therapy to Ameliorate Complications of COVID-19 (ATTACC) trial) failed to show clinical benefits with therapeutic versus standard dose and was prematurely terminated due to the prespecified futility criteria [9]. Interestingly, in the study derived by the same platforms but including non-critically ill patients, a significant clinical benefit was observed for patients receiving therapeutic doses [10]. In the latter trials, patients were randomly assigned to receive therapeutic dose of anticoagulation with unfractionated heparin or LMWH or to receive usual-care pharmacologic thromboprophylaxis which included either standard low dose or enhanced intermediate dose of thromboprophylaxis [9,10]. In another recent randomized clinical trial, therapeutic dose of LMWH reduced major thromboembolism and death compared with institutional standard prophylactic or intermediate dose of LMWH or unfractionated heparin among hospitalized patients with COVID-19 with very elevated D-dimer levels, but interestingly this treatment effect was not evident in ICU patients [13]. It should be noted that the above-mentioned studies comparing therapeutic versus standard dose of thromboprophylaxis were not included in the present meta-analysis because both prophylactic and intermediate doses were used in the standard arm. However, all these findings support a benefit in favor of a more intensive thromboprophylaxis when this is administered early in selected patients with adverse prognostic factors and before the advent of critical disease. 

In the present meta-regression analysis, there was a trend for an inverse association between the observed benefit with intermediate versus prophylactic dose of thromboprophylaxis and the percentage of ICU patients. This was additionally confirmed in sensitivity analyses, including studies conducted exclusively in ICU, compared with general wards or mixed settings (general ward/ICU). This is in line with previous observations and highlights the important issue of the prompt initiation of thromboprophylaxis. However, this observation was not valid for therapeutic versus prophylactic dose of thromboprophylaxis and could be attributed to the ecological bias of the meta-regression analysis. Unfortunately, data regarding the time of initiation of thromboprophylaxis in relation to symptoms onset were not available in the majority of included studies.

An interesting finding in the present analysis was that no difference in the risk for VTE was observed for higher versus prophylactic dose of thromboprophylaxis. However, all these analyses regarded unadjusted estimates and details on the screening or the diagnostic algorithm strategies for VTE were absent. Indeed, the VTE rate differs considerably among the studies, with higher rates among studies implementing universal screening [2]. Thus, minor VTE might be uncaptured in most of the studies. Furthermore, LMWH, apart from its anticoagulant action, has anti-inflammatory effects, which might justify its beneficial role in terms of mortality, above and beyond simply reducing VTE [44,45].

The issue of safety is of paramount importance. This analysis confirmed a higher risk of major bleeding events with therapeutic versus prophylactic dose of thromboprophylaxis, whereas this was not valid for the intermediate dose. However, these analyses were unadjusted and patients with critical disease are frail with complex hematological dysregulations and at risk for complications. In the REMAP-CAP, ACTIV-4a, and ATTACC trial with critically ill patients, hemorrhagic events were more common in patients receiving therapeutic dose compared with the standard arm [9].

Two relevant meta-analyses have been identified through our literature search [46,47]. Both of them analyzed studies comparing the efficacy and safety of therapeutic versus prophylactic dose of thromboprophylaxis and confirmed a trend towards clinical benefits of therapeutic dose. However, mixed adjusted and unadjusted estimates were used, rendering these analyses inconclusive [46,47]. In the present meta-analysis, only high-quality studies with adjusted effect size estimates were included. Moreover, to the best of our knowledge, this is the first time that a comparison between intermediate and prophylactic dose has been performed.

The findings of this analysis should be examined in light of the fact that the available evidence was derived from studies with high heterogeneity regarding the patients’ characteristics, as well as the treatment strategies applied. Combining estimates from different types of studies can be problematic, but it should be mentioned that this meta-analysis applied strict methodological criteria and included studies with high quality. Furthermore, the performance of RR in studies with high mortality rates can be challenging, but meta-regression and sensitivity analyses confirmed the consistency of our findings across heterogeneous studies. Moreover, a small study effect was observed in the comparison of the intermediate versus prophylactic dose. Yet, it should be mentioned that when fewer than 10 studies are included in the meta-analysis, the power of the test for funnel plot asymmetry is too low to distinguish chance from asymmetry. Lastly, the definition of the intensity of thromboprophylaxis might differ among studies with the implemented protocols adjusted for weight and creatinine clearance. For example, the dose of LMWH might be escalated in obese patients but can still be regarded as standard prophylactic dose.

## 5. Conclusions

Evidence on the optimal thromboprophylaxis for hospitalized patients with COVID-19 is derived mainly from observational studies with significant methodological limitations. This meta-analysis of randomized and non-randomized studies with adjusted analyses showed a potentially beneficial impact of enhanced intensity of thromboprophylaxis compared with the standard one. Thus, higher than prophylactic doses of thromboprophylaxis, mainly in the context of an intermediate dose, can be considered for selected patients with COVID-19 at high thrombotic risk. In addition, prompt initiation of thromboprophylaxis appears to be as important as the optimal dose. Randomized trials with strict methodological criteria are needed to provide the highest level of evidence.

## Figures and Tables

**Table 1:** Main characteristics of included studies that compared intermediate or therapeutic versus prophylactic dose of thromboprophylaxis in terms of outcomes in hospitalized COVID-19 patients.

Study	Design	N	ICU (%)	Males (%)	I/P or T/P (%)	Type of Anticoagulation
***Intermediate* versus *prophylactic dose***						
Peperu et al. [30]	R	173	62	56	50/50	LMWH
Sadeghipour et al. [11]	R	562	100	58	49/51	LMWH/UFH
Jimenez-Soto et al. [29]	O	244	0	66	55/45	LMWH
Jonmarker et al. [35]	O	115	100	82	42/58	LMWH
Hsu et al. [34]	O	393	NR	55	4/96	LMWH/UFH/DOAC/VKA
Paolisso et al. [28]	O	450	0	63	20/80	LMWH
Stessel et al. [27]	O	72	100	68	36/64	LMWH
***Therapeutic* versus *prophylactic dose***						
Lopes et al. [33]	R	615	6	60	51/49	LMWH/DOAC
Lemos et al. [12]	R	20	100	80	50/50	LMWH/UFH
Matli et al. [31]	O	82	0	62	38/62	LMWH/UFH/DOAC/Fondaparinux
Copur et al. [32]	O	115	0	50	40/60	LMWH
Jimenez-Soto et al. [29]	O	186	0	67	41/59	LMWH
Roomi et al. [26]	O	176	NR	NR	19/81	NR
Di Castelnuovo et al. [41]	O	1577	NR	NR	30/70	UFH
Motta et al. [25]	O	374	17	59	20/80	LMWH/UFH
Canoglu et al. [40]	O	154	NR	62	36/64	LMWH
Jonmarker et al. [35]	O	104	100	87	36/64	LMWH
Bolzetta et al. [39]	O	81	0	60	30/70	LMWH/UFH/Fondaparinux
Lynn et al. [38]	O	402	27	54	38/62	LMWH/UFH/DOAC
Ionescu et al. [24]	O	3119	20	49	32/68	LMWH/UFH/DOAC/VKA
Hsu et al. [34]	O	425	NR	55	11/89	LMWH/UFH/DOAC/VKA
Ferguson et al. [37]	O	141	100	55	33/67	LMWH/UFH
Secco et al. [23]	O	112	NR	70	43/57	LMWH/DOAC/VKA/Fondaparinux
Bousquet et al. [36]	O	93	0	NR	34/66	NR

DOAC, direct oral anticoagulants; I, intermediate dose; LMWH, low molecular weight heparin; NR, not reported; O, observational; P, prophylactic dose; R, randomized; T, therapeutic dose; UFH, unfractionated heparin; VKA, vitamin K antagonists.

## Data Availability

The data that support the findings of this study are available from the corresponding author (A.K.) upon reasonable request.

## Supplementary Materials

The following are available online at https://www.mdpi.com/article/10.3390/jcm10235549/s1, Figure S1: The PRISMA 2020 flow diagram for systematic reviews and meta-analyses study selection, Figure S2: Begg’s funnel plot for the assessment of publication bias of included studies comparing (**a**) intermediate versus prophylactic and (**b**) therapeutic versus prophylactic thromboprophylaxis dose, Table S1: The PRISMA 2020 for Abstracts Checklist for the present meta-analysis, Table S2: The PRISMA 2020 for Abstracts Checklist for the present meta-analysis, Table S3: List of adjustment variables regarding the included observational studies, Table S4: The assessment of the risk of bias of the included observational studies (comparing intermediate versus prophylactic dose) for the present meta-analysis using a checklist from Joanna Briggs Institute Critical Appraisal Checklists for Cohort Studies, Table S5: The assessment of the risk of bias of the included observational studies (comparing therapeutic versus prophylactic dose) for the present meta-analysis using a checklist from Joanna Briggs Institute Critical Appraisal Checklists for Cohort Studies, Table S6: The assessment of the risk of bias of the included randomized clinical trials in the present meta-analysis using a checklist from Joanna Briggs Institute Critical Appraisal Checklists for Randomized Clinical Trials, Table S7: Certainty of the evidence on the outcome of death for the present meta-analysis using the GRADE approach.
