# Supplementary material for: High versus Standard Intensity of Thromboprophylaxis in Hospitalized Patients with COVID-19: A Systematic Review and Meta-Analysis
Source: J Clin Med. 2021 Nov 26;10(23):5549. doi: 10.3390/jcm10235549 (PMC8658422; doi:10.3390/jcm10235549)
Supplement: Supplementary file 1 [file jcm-10-05549-s001.zip › jcm-1461868 supplementary.pdf]

High Versus Standard Intensity of Thromboprophylaxis in Hospitalized COVID-19 Patients and Outcome: A Systematic Review and Meta-Analysis

SUPPLEMENTARY FILE

Figure S1 The PRISMA 2020 flow diagram for systematic reviews and meta-analyses study selection

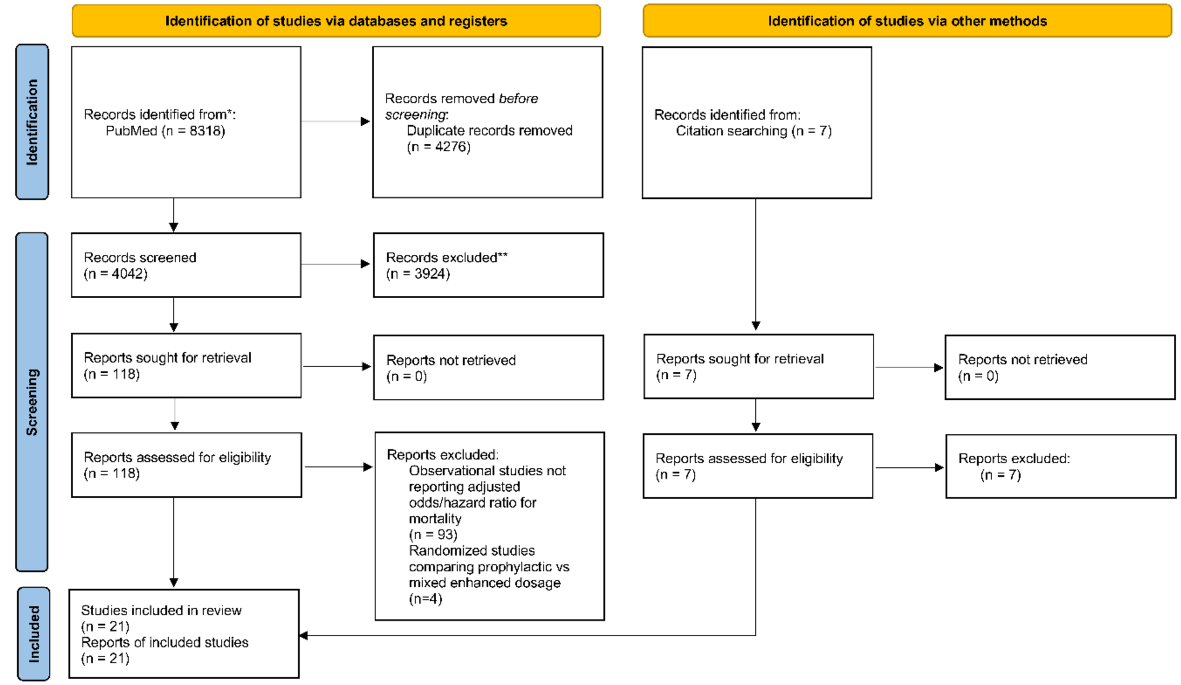

*Consider, if feasible to do so, reporting the number of records identified from each database or register searched (rather than the total number across all databases/registers).
**If automation tools were used, indicate how many records were excluded by a human and how many were excluded by automation tools.

From: Page MJ, McKenzie JE, Bossuyt PM, Boutron I, Hoffmann TC, Mulrow CD, et al. The PRISMA 2020 statement: an updated guideline for reporting systematic reviews. BMJ 2021;372:n71. doi: 10.1136/bmj.n71. For more information, visit: http://www.prisma-statement.org/

Figure S2 Begg's funnel plot for the assessment of publication bias of included studies comparing (a) intermediate versus prophylactic and (b) therapeutic versus prophylactic dose of thromboprophylaxis

(a)

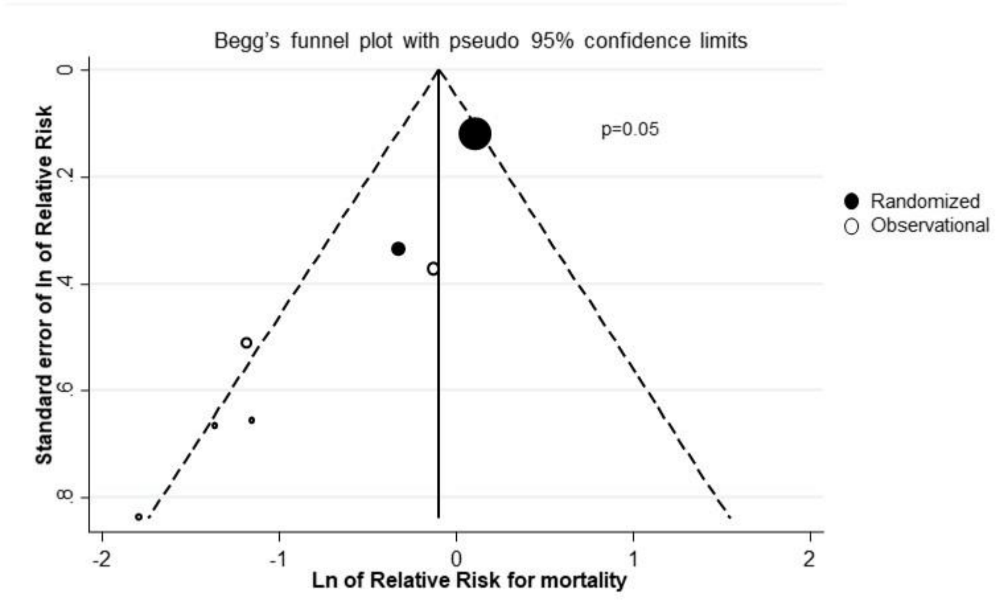

(b)

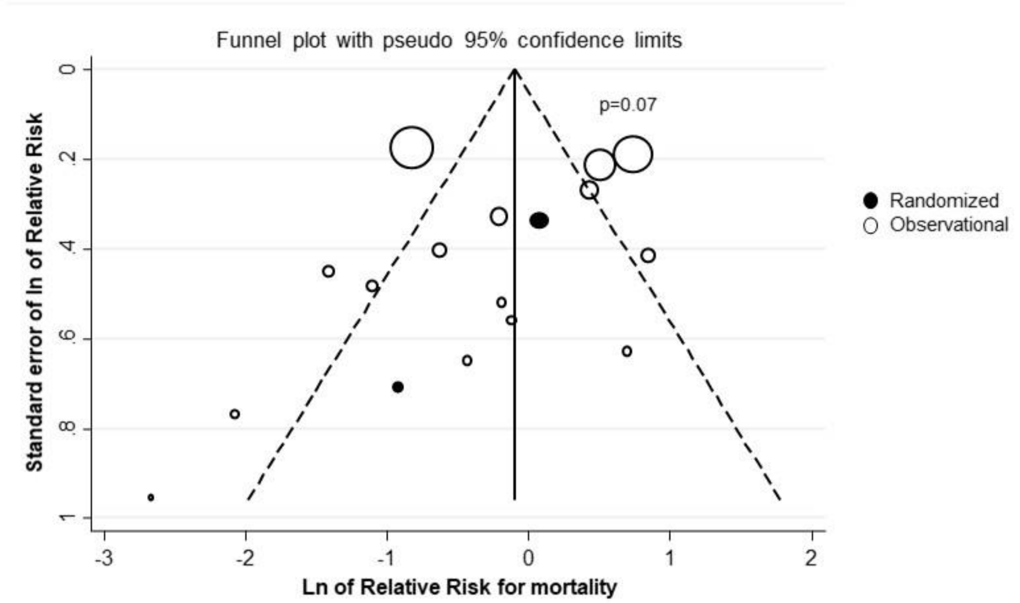

Table S1. The PRISMA 2020 Checklist for the present meta-analysis.

Section and Topic	Item #	Checklist item	Location where item is reported
**TITLE**			
Title	1	Identify the report as a systematic review.	Page 1, Title
**ABSTRACT**			
Abstract	2	See the PRISMA 2020 for Abstracts checklist.	Table S2
**INTRODUCTION**			
Rationale	3	Describe the rationale for the review in the context of existing knowledge.	Page 1, 2
Objectives	4	Provide an explicit statement of the objective(s) or question(s) the review addresses.	Page 2
**METHODS**			
Eligibility criteria	5	Specify the inclusion and exclusion criteria for the review and how studies were grouped for the syntheses.	Page 2
Information sources	6	Specify all databases, registers, websites, organisations, reference lists and other sources searched or consulted to identify studies. Specify the date when each source was last searched or consulted.	Page 2
Search strategy	7	Present the full search strategies for all databases, registers and websites, including any filters and limits used.	Page 2
Selection process	8	Specify the methods used to decide whether a study met the inclusion criteria of the review, including how many reviewers screened each record and each report retrieved, whether they worked independently, and if applicable, details of automation tools used in the process.	Page 2
Data collection process	9	Specify the methods used to collect data from reports, including how many reviewers collected data from each report, whether they worked independently, any processes for obtaining or confirming data from study investigators, and if applicable, details of automation tools used in the process.	Page 2
Data items	10a	List and define all outcomes for which data were sought. Specify whether all results that were compatible with each outcome domain in each study were sought (e.g. for all measures, time points, analyses), and if not, the methods used to decide which results to collect.	Page 2, 3
	10b	List and define all other variables for which data were sought (e.g. participant and intervention characteristics, funding sources). Describe any assumptions made about any missing or unclear information.	Page 3
Study risk of bias assessment	11	Specify the methods used to assess risk of bias in the included studies, including details of the tool(s) used, how many reviewers assessed each study and whether they worked independently, and if applicable, details of automation tools used in the process.	Page 3
Effect measures	12	Specify for each outcome the effect measure(s) (e.g. risk ratio, mean difference) used in the synthesis or presentation of results.	Page 3
Synthesis methods	13a	Describe the processes used to decide which studies were eligible for each synthesis (e.g. tabulating the study intervention characteristics and comparing against the planned groups for each synthesis (item #5)).	Page 3
	13b	Describe any methods required to prepare the data for presentation or synthesis, such as handling of missing summary statistics, or data conversions.	Page 3
	13c	Describe any methods used to tabulate or visually display results of individual studies and syntheses.	Page 3
	13d	Describe any methods used to synthesize results and provide a rationale for the choice(s). If meta-analysis was performed, describe the model(s), method(s) to	Page 3

Section and Topic	Item #	Checklist item	Location where item is reported
		identify the presence and extent of statistical heterogeneity, and software package(s) used.	
	13e	Describe any methods used to explore possible causes of heterogeneity among study results (e.g. subgroup analysis, meta-regression).	Page 3
	13f	Describe any sensitivity analyses conducted to assess robustness of the synthesized results.	Page 3
Reporting bias assessment	14	Describe any methods used to assess risk of bias due to missing results in a synthesis (arising from reporting biases).	Page 3
Certainty assessment	15	Describe any methods used to assess certainty (or confidence) in the body of evidence for an outcome.	Page 3
**RESULTS**			
Study selection	16a	Describe the results of the search and selection process, from the number of records identified in the search to the number of studies included in the review, ideally using a flow diagram.	Page 3
	16b	Cite studies that might appear to meet the inclusion criteria, but which were excluded, and explain why they were excluded.	Page 3, Figure S1
Study characteristic s	17	Cite each included study and present its characteristics.	Page 3-5, Table 1
Risk of bias in studies	18	Present assessments of risk of bias for each included study.	Page 6
Results of individual studies	19	For all outcomes, present, for each study: (a) summary statistics for each group (where appropriate) and (b) an effect estimate and its precision (e.g. confidence/credible interval), ideally using structured tables or plots.	Page 4, 5, Table 1, Table S3
Results of syntheses	20a	For each synthesis, briefly summarise the characteristics and risk of bias among contributing studies.	Page 4-6
	20b	Present results of all statistical syntheses conducted. If meta-analysis was done, present for each the summary estimate and its precision (e.g. confidence/credible interval) and measures of statistical heterogeneity. If comparing groups, describe the direction of the effect.	Page 4-6
	20c	Present results of all investigations of possible causes of heterogeneity among study results.	Page 4-6
	20d	Present results of all sensitivity analyses conducted to assess the robustness of the synthesized results.	Page 6
Reporting biases	21	Present assessments of risk of bias due to missing results (arising from reporting biases) for each synthesis assessed.	Page 6, Figure S2, Table S4, S5, S6
Certainty of evidence	22	Present assessments of certainty (or confidence) in the body of evidence for each outcome assessed.	Page 6, Table S7
**DISCUSSION**			
Discussion	23a	Provide a general interpretation of the results in the context of other evidence.	Page 6, 7
	23b	Discuss any limitations of the evidence included in the review.	Page 8, 9
	23c	Discuss any limitations of the review processes used.	Page 8, 9
	23d	Discuss implications of the results for practice, policy, and future research.	Page 8, 9
**OTHER INFORMATION**			
Registration and protocol	24a	Provide registration information for the review, including register name and registration number, or state that the review was not registered.	Page 2
	24b	Indicate where the review protocol can be accessed, or state that a protocol was not prepared.	Page 2

Section and Topic	Item #	Checklist item	Location where item is reported
	24c	Describe and explain any amendments to information provided at registration or in the protocol.	Page 2
Support	25	Describe sources of financial or non-financial support for the review, and the role of the funders or sponsors in the review.	Page 9
Competing interests	26	Declare any competing interests of review authors.	Page 9
Availability of data, code and other materials	27	Report which of the following are publicly available and where they can be found: template data collection forms; data extracted from included studies; data used for all analyses; analytic code; any other materials used in the review.	Page 9

From: Page MJ, McKenzie JE, Bossuyt PM, Boutron I, Hoffmann TC, Mulrow CD, et al. The PRISMA 2020 statement: an updated guideline for reporting systematic reviews. BMJ 2021;372:n71. doi: 10.1136/bmj.n71

For more information, visit: http://www.prisma-statement.org/

Table S2. The PRISMA 2020 for Abstracts Checklist for the present meta-analysis.

Section and Topic	Item #	Checklist item	Reported (Yes/No)
		**TITLE**	
Title	1	Identify the report as a systematic review.	Yes
		**BACKGROUND**	
Objectives	2	Provide an explicit statement of the main objective(s) or question(s) the review addresses.	Yes
		**METHODS**	
Eligibility criteria	3	Specify the inclusion and exclusion criteria for the review.	Yes
Information sources	4	Specify the information sources (e.g. databases, registers) used to identify studies and the date when each was last searched.	Yes
Risk of bias	5	Specify the methods used to assess risk of bias in the included studies.	Yes
Synthesis of results	6	Specify the methods used to present and synthesise results.	Yes
		**RESULTS**	
Included studies	7	Give the total number of included studies and participants and summarise relevant characteristics of studies.	Yes
Synthesis of results	8	Present results for main outcomes, preferably indicating the number of included studies and participants for each. If meta-analysis was done, report the summary estimate and confidence/credible interval. If comparing groups, indicate the direction of the effect (i.e. which group is favoured).	Yes
		**DISCUSSION**	
Limitations of evidence	9	Provide a brief summary of the limitations of the evidence included in the review (e.g. study risk of bias, inconsistency and imprecision).	Yes
Interpretation	10	Provide a general interpretation of the results and important implications.	Yes
		**OTHER**	
Funding	11	Specify the primary source of funding for the review.	Yes
Registration	12	Provide the register name and registration number.	Yes

From: Page MJ, McKenzie JE, Bossuyt PM, Boutron I, Hoffmann TC, Mulrow CD, et al. The PRISMA 2020 statement: an updated guideline for reporting systematic reviews. BMJ 2021;372:n71. doi: 10.1136/bmj.n71

For more information, visit: http://www.prisma-statement.org/

Table S3 List of adjustment variables regarding the included observational studies

Study	Adjustment variables
	Intermediate vs prophylactic dose
Jimenez-Soto et al	Age, sex, c-reactive protein, D-dimer, hypertension, invasive mechanical ventilation
Jonmarker et al	Age, sex, body mass index, invasive mechanical ventilation, Simplified Acute Physiology Score III
Hsu et al	Age, sex, indicators of COVID-19 severity, baseline comorbidities
Paolisso et al	Age, hypertension, hemoglobin, PO_2/FiO_2, hydroxychloroquine, tocilizumab
Stessel et al	Age, sex, body mass index, hypertension, diabetes, SOFA and Apache II score, D-dimer, white blood cell count
	Therapeutic vs prophylactic dose
Matli et al	Age, sex, smoking, weight, hypertension, dyslipidemia, diabetes, coronary artery disease, steroids, tofacitinib, immunosuppressant, antiplatelet use on admission, anticoagulation and antiplatelet regimen, inpatient prescription of any of the following medication: azithromycin, fondaparinux, remdesivir, tocilizumab, oxygen saturation on admission, D-dimer, c-reactive protein, interleukin 6
Copur et al	Age, sex, c-reactive protein, National Early Warning Score 2
Jimenez-Soto et al	Age, sex, c-reactive protein, D-dimer, hypertension, invasive mechanical ventilation
Roomi et al	Age, sex, diabetes, hypertension, coronary artery disease, chronic kidney disease, chronic obstructive pulmonary disease, prior anticoagulation, use of hydroxychloroquine, tocilizumab, remdesivir, steroids
Di Castelnuovo et al	Age, sex, diabetes, hypertension, ischemic heart disease, chronic pulmonary disease, chronic kidney disease, c-reactive protein, hydroxychloroquine, other in-hospital therapies
Motta et al	Age, ethnicity, diabetes, cancer, heart disease, intensive care, mechanical ventilation, antibiotics, hydroxychloroquine, tocilizumab
Canoglu et al	Not reported
Jonmarker et al	Age, sex, body mass index, invasive mechanical ventilation, Simplified Acute Physiology Score III
Bolzetta et al	Age, sex, obesity, diabetes, cancer, chronic obstructive pulmonary disease, dementia, Parkison's disease, renal failure, acute myocardial infarction, stroke, heart failure, high blood pressure, pressure sores, hepatic cirrhosis
Lynn et al	Not reported
Ionescu et al	Age, sex, race (Caucasian, African American, Asian, Other), body mass index, hypertension, hyperlipidemia, coronary artery disease, peripheral artery disease, heart failure, cerebral vascular attack/transient ischemic attack, atrial fibrillation, chronic kidney disease grade 3 or above,

	hemodialysis, malignancy, venous thromboembolism, immunocompromised status, connective tissue disease, chronic lung disease
Hsu et al	Age, sex, indicators of COVID-19 severity, baseline comorbidities
Ferguson et al	Adjuvant treatment
Secco et al	Not reported
Bousquet et al	Activity of Daily Living score, muscle weakness (yes/no), Mini Geriatric Depression Scale, D-dimer, lactate dehydrogenase

Table S4. The assessment of the risk of bias of the included observational studies comparing intermediate versus prophylactic dosage for the present meta-analysis using a checklist from Joanna Briggs Institute Critical Appraisal Checklists for Cohort Studies.

		**Jimenez-Soto et al**	**Jonmarker et al**	**Hsu et al**	**Paolisso et al**	**Stessel et al**
Q1	Were the two groups similar and recruited from the same population?	U	U	N	N	N
Q2	Were the exposures measured similarly to assign people to both exposed and unexposed groups?	Y	Y	Y	Y	Y
Q3	Was the exposure measured in a valid and reliable way?	Y	Y	Y	Y	Y
Q4	Were confounding factors identified?	Y	Y	Y	Y	Y
Q5	Were strategies to deal with confounding factors stated?	Y	Y	Y	Y	Y
Q6	Were the groups/participants free of the outcome at the start of the study (or at the moment of exposure)?	Y	Y	Y	Y	Y
Q7	Were the outcomes measured in a valid and reliable way?	Y	Y	Y	Y	Y
Q8	Was the follow up time reported and sufficient to be long enough for outcomes to occur?	U	Y	Y	U	Y
Q9	Was follow up complete, and if not, were the reasons to loss to follow up described and explored?	Y	Y	Y	Y	Y
Q10	Were strategies to address incomplete follow up utilized?	Y	Y	Y	Y	Y
Q11	Was appropriate statistical analysis used?	Y	Y	Y	Y	Y
Grading		10	10,5	10	9,5	10
Risk of Bias		Low	Low	Low	Low	Low

N, no; U, unclear; Y, yes. For studies grading, 'Yes' was graded with 1, 'Unclear' with 0.5, 'No' with 0; Studies with ≥8 'Yes' were categorized as of low risk of bias; Clarifications for Q1 and Q8: Q1: groups were recruited from the same population, but in order to be considered similar there should be no differences in the following baseline characteristics: age, gender distribution, body mass index and ratio of partial pressure arterial oxygen and fraction of inspired oxygen (PaO_2/FiO_2) or oxygen saturation. Q8: follow-up was considered sufficient if it was ≥ 14 days.

Table S5. The assessment of the risk of bias of the included observational studies comparing therapeutic versus prophylactic dosage for the present meta-analysis using a checklist from Joanna Briggs Institute Critical Appraisal Checklists for Cohort Studies.

		**Matli et al**	**Copur et al**	**Jimenez-Soto et al**	**Roomi et al**	**Di Castelnuovo et al**	**Motta et al**	**Canoglu et al**	**Jonmarker et al**	**Bolzetta et al**	**Lynn et al**	**Ionescu et al**	**Hsu et al**	**Ferguson et al**	**Secco et al**	**Bousquet et al**
Q1	Were the two groups similar and recruited from the same population?	N	N	U	U	U	N	U	N	N	U	U	U	U	U	U
Q2	Were the exposures measured similarly to assign people to both exposed and unexposed groups?	Y	Y	Y	Y	Y	Y	Y	Y	Y	Y	Y	Y	Y	Y	Y
Q3	Was the exposure measured in a valid and reliable way?	Y	Y	Y	Y	Y	Y	Y	Y	Y	Y	Y	Y	Y	Y	Y
Q4	Were confounding factors identified?	Y	Y	Y	Y	Y	Y	Y	Y	Y	Y	Y	Y	Y	Y	Y
Q5	Were strategies to deal with confounding factors stated?	Y	Y	Y	Y	Y	Y	Y	Y	Y	Y	Y	Y	Y	Y	Y
Q6	Were the groups/participants free of the outcome at the start of the study (or at the moment of exposure)?	Y	Y	Y	Y	Y	Y	Y	Y	Y	Y	Y	Y	Y	Y	Y
Q7	Were the outcomes measured in a valid and reliable way?	Y	Y	Y	Y	Y	Y	Y	Y	Y	Y	Y	Y	Y	Y	Y
Q8	Was the follow up time reported and sufficient to be long enough for outcomes to occur?	U	U	U	U	Y	U	U	N	U	Y	Y	Y	U	U	N
Q9	Was follow up complete, and if not, were the reasons to loss to follow up described and explored?	Y	Y	Y	Y	Y	Y	Y	Y	Y	Y	Y	Y	Y	Y	Y
Q10	Were strategies to address incomplete follow up utilized?	Y	Y	Y	Y	Y	Y	Y	Y	Y	Y	Y	Y	Y	Y	Y
Q11	Was appropriate statistical analysis used?	Y	Y	Y	Y	Y	Y	Y	Y	Y	Y	Y	Y	Y	Y	Y
Grading		9,5	9,5	10	10	10,5	9,5	10	10	9,5	10,5	10,5	10,5	10	10	9,5
Risk of Bias		Low	Low	Low	Low	Low	Low	Low	Low	Low	Low	Low	Low	Low	Low	Low

N, no; U, unclear; Y, yes; For studies grading, ‘Yes’ was graded with 1, ‘Unclear’ with 0.5, ‘No’ with 0; Studies with ≥8 ‘Yes’ were categorized as of low risk of bias; Clarifications for Q1 and Q8: Q1: groups were recruited from the same population, but in order to be considered similar there should be no differences in the following baseline characteristics: age, gender distribution, body mass index and ratio of partial pressure arterial oxygen and fraction of inspired oxygen (PaO_2/FiO_2) or oxygen saturation. Q8: follow-up was considered sufficient if it was ≥ 14 days.

Table S6. The assessment of the risk of bias of the included randomized clinical trials in the present meta-analysis using a checklist from Joanna Briggs Institute Critical Appraisal Checklists for Randomized Clinical Trials.

Comparison arms		Intermediate versus Prophylactic dose		Therapeutic versus Prophylactic dose	
		Perepu et al	Sadeghipour et al	Lopes et al	Lemos et al
Q1	Was true randomization used for assignment of participants to treatment groups?	Y	Y	Y	Y
Q2	Was allocation to treatment groups concealed?	U	Y	Y	Y
Q3	Were treatment groups similar at the baseline?	Y	Y	Y	Y
Q4	Were participants blind to treatment assignment?	N	N	N	N
Q5	Were those delivering treatment blind to treatment assignment?	N	N	N	N
Q6	Were outcomes assessors blind to treatment assignment?	N	Y	Y	Y
Q7	Were treatment groups treated identically other than the intervention of interest?	Y	Y	Y	Y
Q8	Was follow up complete and if not, were differences between groups in terms of their follow up adequately described and analyzed?	Y	Y	Y	Y
Q9	Were participants analyzed in the groups to which they were randomized?	Y	Y	Y	Y

Q10	Were outcomes measured in the same way for treatment groups?	Y	Y	Y	Y
Q11	Were outcomes measured in a reliable way?	Y	Y	Y	Y
Q12	Was appropriate statistical analysis used?	Y	Y	Y	Y
Q13	Was the trial design appropriate, and any deviations from the standard RCT design (individual randomization, parallel groups) accounted for in the conduct and analysis of the trial?	Y	Y	Y	Y
Grading		9,5	11	11	11
Risk of Bias		Low	Low	Low	Low

N, no; U, unclear; Y, yes; For studies grading, 'Yes' was graded with 1, 'Unclear' with 0.5, 'No' with 0; Studies with ≥9 'Yes' were categorized as of low risk of bias

Table S7. Certainty of the evidence on the outcome of death for the present meta-analysis using the GRADE approach.

Domains for assessing certainty of evidence by outcome	Certainty of evidence
Risk of bias	Not downgraded. Despite some concerns (most studies of non-randomized design), the selection criteria for the non-randomized studies included that the outcome of interest would be adjusted for confounders. Thus, all studies were deemed as low risk of bias.
Inconsistency	Downgraded. The heterogeneity was important ($I^2 > 50\%$).
Indirectness	Not downgraded. The included studies were restricted to hospitalized patients with COVID-19.
Imprecision	Downgraded. The confidence intervals for the effect on mortality were considerably wide in most of the included studies.
Publication bias	Downgraded. A small study effect was revealed for intermediate versus prophylactic dose.
Large effects (upgrading)	Not upgraded. The pooled relative risk was > 0.5.
Dose response (upgrading)	Not upgraded. Benefit was observed with intermediate but not with therapeutic dosage.
Opposing plausible residual bias and confounding (upgrading)	Upgraded. Taking into consideration that more aggressive therapies are administered in more critical disease and that in some of the included studies there were differences in baseline characteristics of the comparison groups (usually not in favor of the enhanced dosage groups), opposing plausible residual bias might be present.
